# Cross-sectoral video consultation in cancer care: GPs’ evaluation of a randomised controlled trial

**DOI:** 10.3399/BJGPO.2020.0114

**Published:** 2021-02-17

**Authors:** Theis Bitz Trabjerg, Lars Henrik Jensen, Jens Sondergaard, Sonja Wehberg, Jeffrey James Sisler, Dorte Gilså Hansen

**Affiliations:** 1 Research Unit of General Practice, Department of Public Health, University of Southern Denmark, Odense, Denmark; 2 Department of Oncology, Lillebælt Hospital, University Hospital of Southern Denmark, Vejle, Denmark; 3 Department of Regional Health Research, Lillebælt Hospital, Center of Clinical Excellence, Danish Colorectal Cancer Center South, University Hospital of Southern Denmark, Vejle, Denmark; 4 Department of Family Medicine, Faculty of Health Sciences, University of Manitoba, Winnipeg, MB, Canada; 5 Center for Shared Decision Making, Lillebælt Hospital, University Hospital of Southern Denmark, Vejle, Denmark

**Keywords:** Cancer, shared care, telemedicine, general practice, patient reported outcome measures

## Abstract

**Background:**

Shared care models present an opportunity for patients to receive the benefits of specialist care combined with the continuity of care provided by a GP.

**Aim:**

To test the effects on GP-perceived involvement in cancer care and their satisfaction with this cross-sectoral information after bringing the patient, GP, and oncologist together in a shared video consultation.

**Design & setting:**

GPs from the Region of Southern Denmark evaluated a randomised controlled trial testing shared video consultations.

**Method:**

This study describes secondary outcomes based on a 4 months' follow-up survey from GPs participating in The Partnership Project (PSP). Patient perception of coordination of care at 7 months' follow-up was the primary outcome of the PSP. A tripartite video consultation was conducted during cancer treatment to share tasks and roles between health professionals with the patient.

**Results:**

The study included 281 patients, and 105 unique GPs returned 124 questionnaires. Video consultations were accomplished in 68% of scheduled cases. The study found an increased odds ratio (OR) of 3.03 for GP satisfaction with the distribution of tasks and roles, and they experienced more involvement in the cancer patients' trajectory. The study found an increased OR of 6.95 for the GP perception of more direct contact and dialogue with the Department of Oncology. There was a decreased OR of 0.88 for the GP to be engaged in handling anxiety and psychological concerns.

**Conclusion:**

The study showed that involving the GP in one shared consultation increased the odds of the GP being satisfied with the distribution of tasks and roles, and feeling more involved in the cancer patient’s trajectory. However, recruitment and response rates from GPs were limiting factors.

## How this fits in

Shared video consultation between cancer patient, oncologist, and GP may increase the GP’s perception of information exchange, involvement in cancer care, and clarification of tasks and roles between sectors. This evaluation of secondary outcomes from a randomised controlled trial showed that a 15-minute shared video consultation can increase the GPs' satisfaction and clarification of their task and roles during a cancer trajectory. Future patient-reported outcomes will investigate if this intervention also benefits patients.

## Introduction

More involvement of primary care is suggested to improve the quality of cancer care. Giving the best support during and following long-term cancer treatment poses considerable challenges for GPs.^1–4^ GPs request better communication and collaboration with hospital specialists.^5^ Enhanced involvement by the GP is demanded by cancer patients.^6–8^

To provide effective cancer care, new patient-centred models supporting the exchange of knowledge and task clarification between oncologists and GPs are needed.^9^ Shared care is understood as *‘* *an organizational model involving both primary care physicians and specialists in a formal, explicit manner* *’*.^10^ Different elements are thought to improve treatment quality, like formal communication channels, role clarification, and enhanced patient confidence in GPs skills and competences. Shared care models present an opportunity for patients to receive the benefits of specialist care combined with the continuity of care and management of comorbidity provided by the GP.^11^ This implies a patient-centred outset and the development of a supporting communication model.^12^ Bringing both patient, GP, and oncologist together in a shared consultation might be a powerful solution as an add-on to usual care, consisting of written information between sectors, without involving the cancer patient.

Due to geographical reasons and shortage of health professionals' time, shared consultations are not feasible in daily routine. Video consultations have become increasingly common,^13,14^ and video-based communication may be an alternative solution to connect health professionals.^15–17^ The authors were not aware of studies exploring video consultations bringing a patient together with their GP and oncologist. However, video solutions have been used for multidisciplinary team meetings in cancer treatment planning.^18^ Recent trials have included GPs^19^ or patients,^20,21^ but not simultaneously. Therefore, the authors developed the partnership shared care model, including multidisciplinary video consultations between a cancer patient, oncologist, and GP.^22^ Based on a randomised controlled trial, this study aimed to test the effects on GP perceived involvement in cancer care, and their satisfaction with cross-sectoral information and coordination of care.

## Method

### Study design

This study reported secondary outcomes based on a 4 months' follow-up survey from GPs participating in the randomised controlled trial, PSP.^22^ Inclusion of patients was accomplished between June 2016 and November 2019. Patient perception of coordination of care at 7 months' follow-up was the primary outcome.^22^ This study follows the CONSORT statement^23^ (see Supplementary Table S1).

### Setting

Patients were invited to participate in the study at the Department of Oncology, Lillebælt Hospital, if aged >18 years, treated with chemotherapy for a newly diagnosed cancer, and listed with a GP in the Region of Southern Denmark. GPs were all specialists in family medicine.^22^

Around 1300 cancer patients are referred to chemotherapy at the Department of Oncology, annually. The Region has 500 GPs working in approximately 300 general practices, comprised of 1 to 8 GPs per practice.

### The Partnership Project intervention

The PSP consultation was planned as early as possible within 12 weeks from time of inclusion, corresponding to a maximum of 18 weeks after the first appointment at the department. The consultation was conducted as part of the planned standard programme at the hospital. If the patient chose to be located at the GP’s office, a further consultation was scheduled. Consultations were booked 3 to 6 weeks in advance within regular clinic hours.

Before each consultation, oncologists and GPs received specific information about the aim of the consultation, including a consultation guide (Box 1) with themes that may be relevant. Not all themes were raised in each consultation, but all themes were found relevant and discussed in different consultations.^24^

Box 1 **The consultation guide to GPs and oncologists, including themes potentially relevant for the consultation**

Consultation guide:

- The oncologist acts as chair of the shared video consultation
- The duration of the consultation should be between 10 to 20 minutes
- The oncologist starts by introducing the participants and the purpose of the shared consultation
- Exchange of information between all participants for the benefit of the patient
- Role and tasks clarification between the Department of Oncology and the GP
- The consultations conclude with a summary, in which it is clarified whether a follow-up is needed with the GP or Department of Oncology
- The consultation and its agreements are documented in the hospitals' electronic patient record and sent to the GP, and made available for the patient online

The list of potential themes (not all themes might be relevant for the patient):

- A summary of the patient trajectory
- Patients concerns and desire for the consultation
- Sharing knowledge regarding comorbidity
- Psychosocial resources and needs
- Agreements on who should take care of what and when in the future
- Physical and psychological well-being
- Medicine
- Relatives
- Ability to work
- Late complications and side effects to the treatment

PSP consultations were accomplished through a video link using a virtual meeting room.

The control group received 'usual care', which included an electronic summary letter to the GP after each visit to the Department of Oncology. In case of questions, GPs could always telephone the hospital, and vice versa. Furthermore, patients could freely contact their GP or a specific nurse coordinator at the Department of Oncology.

### Randomisation and blinding

After informed consent and a baseline questionnaire, patients were allocated in a 1:1 ratio using block randomisation. The GPs and oncologists could have patients in both groups.

Neither patients, GPs, nor oncologists in the intervention group were blinded to the patient’s allocation. During enrolment and baseline data, patients and enrolling nurses were not aware of the randomisation. Data analysts were kept blinded. GPs to patients in the control arm were not formally informed before receiving the survey.

### Outcome measures

The authors developed a GP questionnaire,^22^ including the number of patient visits to the GP and 28 items covering five different themes: GP assessment of the contact between the hospital and GP (two items), information from the hospital to the GP (seven items), GP involvement in the trajectory (six items), information from the hospital to help the GP (six items), and GP satisfaction with the distribution of task and roles (seven items) (see Supplementary Table S2). All questionnaires were measured on a 4-point Likert scale and used the following ‘not at all’, ‘little’, ‘partly’, and ‘a lot’, or, for linguistic reasons, ‘very insufficient’, ‘partly insufficient’, ‘satisfactory’, and ‘very satisfactory’. For analysis, answers were dichotomised with two in favour of the intervention, and two in favour of usual care. Finally, GPs were given the option of commenting (data not shown).

### Data collection

For data availability reasons, GPs in the control group were sent the questionnaire by postal service. The intervention group were managed using RedCap^®^,^25^ which automatically emailed secured links with the surveys. Due to Danish data protection laws, it was not possible to retrain demographic information on GPs who declined to participate or were non-responders.

### Sample size

**Figure 1.**
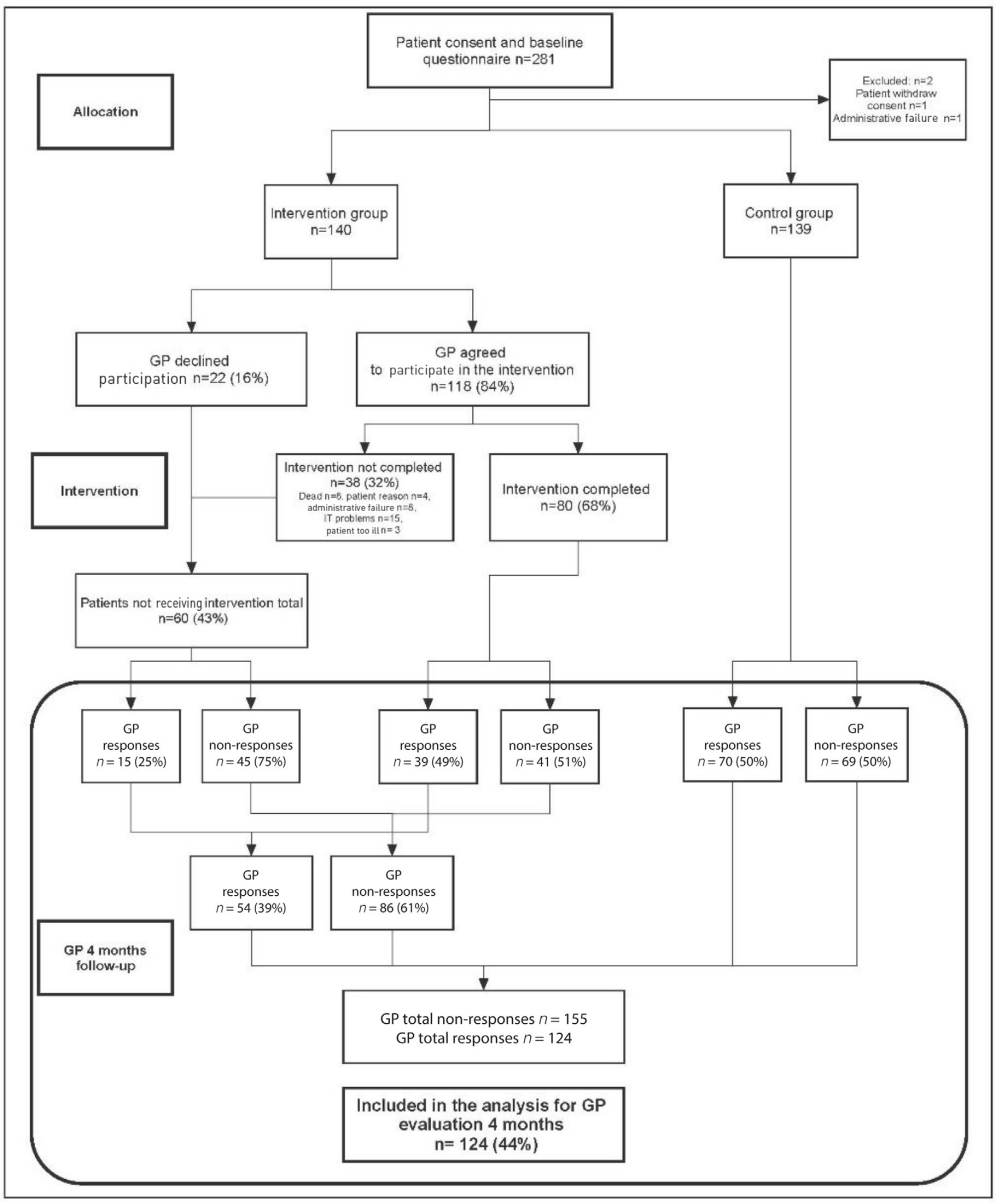
Study flow.

The number of GPs invited was based on sample size calculations, expressly the primary, patient-reported outcome.^22^ The goal was to include 278 patients (Figure 1).

### Statistical analysis

Before performing the between-group analysis, six of the 29 items were chosen as outcomes. The selection was based on the process evaluations of the PSP,^24,26^ the overall distribution of items with an observed floor and ceiling effect for some items, and comments from the survey (data not shown).

For the between-groups analysis, a sensitivity analysis was performed with intention-to-treat and per-protocol analysis. GPs in the intervention group who participated in a video consultation as intended (with video and sound) were defined as having completed per-protocol.

The authors estimated the effect of group status in univariable logistic regression models, taking clustering by GP into account, and presented the results as OR.

Missing values were not imputed. Corrections for multiple testing were not employed. Due to the relatively small sample size, the authors chose not to fit multivariable models.

A *P*-value <0.05 was considered statistically significant.

## Results

Demographic data on patients and GPs are shown in Tables 1 and 2.

**Table 1.** Baseline patient characteristic of the total patient population and the groups based on 105 unique GPs' responses represented by GP responses and non-responses, respectively

**Patient characteristic**	**All (*n* = 279), *n* (%**)	**GP non-responses** **(*n* = 154), *n* (%**)	**GP responses (*n* = 124), *n* (%**)
**Mean age, years (SD** **)**	65.3 (10.6)	65.7 (10.8)	64.8 (10.3)
**Sex**			
Male	155 (55.6)	90 (58.1)	65 (52.4)
Female	124 (44.4)	65 (41.9)	59 (47.6)
**Education**			
Primary school	161 (57.7)	86 (55.8)	75 (60.5)
Upper secondary school	16 (5.7)	10 (6.5)	6 (4.8)
Further education	76 (27.2)	48 (31.0)	28 (22.6)
Higher education	16 (5.7)	6 (3.9)	10 (8.1)
Missing	10 (3.6)	4 (32.6)	5 (4.0)
**Marital status**			
Single	80 (28.7)	46 (29.7)	34 (27.4)
Married/living with someone	198 (71.0)	108 (69.7)	90 (72.6)
**Children at home**			
No	244 (87.5)	134 (86.5)	110 (88.7)
Yes	34 (12.2)	20 (12.9)	14 (11.3)
**Work status**			
Employed	89 (31.9)	50 (32.3)	39 (31.5)
Public benefits	15 (5.4)	9 (5.8)	6 (4.8)
Retired	174 (62.4)	95 (61.3)	79 (63.7)
**Comorbidity**			
No	132 (47.3)	80 (51.9)	51 (41.1)
Yes	147 (52.7)	74 (47.7)	73 (58.9)
**Diagnosis/cancer type**			
Breast	33 (11.8)	17 (11.0)	16 (12.9)
Gynaecological	13 (4.7)	6 (3.9)	7 (5.6)
Lung	106 (38.0)	64 (41.6)	42 (33.9)
Gastrointestinal	111 (39.8)	58 (37.7)	53 (42.7)
Other	16 (5.7)	9 (5.8)	6 (4.8)

Percentages do not come to 100% in all categories due to missing data. However, there a too few missing data to report due to data security.

**Table 2.** Baseline characteristic of GPs participating in the survey (responders)

**GP variables**	**All (*n* = 124), *n* (%**)	**Intervention group** **(*n* = 56), *n* (%**)	**Control group (*n* = 68), *n* (%**)
**Sex**			
Male	61 (49.2)	29 (51.8)	32(47.1)
Female	63 (50.8)	27 (48.2)	36 (52.9)
**Practicing family medicine, years**			
0–9	43 (34.7)	19 (33.9)	24 (35.3)
10–19	32 (25.8)	14 (25.0)	19 (27.9)
≥20	47 (37.9)	23 (41.1)	25 (36.8)
**Full-time GPs in practice**			
1	15 (12.1)	5 (8.9)	10 (14.7)
2	26 (21.0)	11 (19.6)	15 (22.1)
3	29 (23.4)	14 (25.0)	15 (22.1)
4	34 (27.4)	19 (33.9)	15 (22.1)
≥5	20 (16.1)	7 (12.5)	13 (19.1)

In total, 281 patients were included. A total of 124 (44%) questionnaires were returned from 105 unique GPs, eight having patients in both groups and nine GPs paticipated with >1 patient. (Figure 1).

A total of 80 (68%) video consultations were completed as planned (Figure 1). For these, 64 (80%) patients were located at the Department of Oncology, 16 (20%) by the GP. A total of 25 unique oncology specialists had 1 to 13 consultations each.

The number of patient visits to general practice during the 4 months' follow-up was higher among patients in the intervention group than in the control group (data not shown).

GPs’ answers are shown as frequencies in Supplementary Table S2. The regression analysis showed statistically significant OR in favour of the intervention regarding the item *‘* *As a GP, did you experience direct contact or dialogue with the Department of Oncology?* *‘* (OR 6.95, 95% confidence interval [CI] = 2.96 to 16.35) and the item *‘As a GP, how satisfied are you, with the distribution of tasks and roles regarding initiatives practice could initiate concerning the patient trajectory?* *‘* (OR 3.03, 95% CI = 1.35 to 6.82). OR was significant and in the same direction for both intention-to-treat and per-protocol analysis. Regarding the item *‘As a GP, I have been involved in handling the patient’s anxiety and psychological concerns* *‘*, the study found statistically significant decreased odds (OR 0.44, 95% CI = 0.21 to 0.92). The per-protocol analysis resulted in an OR in the same direction, but was insignificant (Table 3).

**Table 3.** Effect of the intervention for the selected six dichotomy items on GPs involvement in different aspects of treatment and GPs satisfaction with information and agreements of roles

		Intention-to-treat	Per-protocol
Items	Responses, *n*	OR (95% CI)^a^	*P* value	OR (95% CI)^a^	*P* value
As a GP, did you experience direct contact or dialogue with the Department of Oncology?	117	6.95 (2.96 to 16.35)	0.000	6.09 (2.64 to 14.03)	0.000
As a GP, I have been involved in the treatment of comorbidity	124	1.35 (0.67 to 2.75)	0.404	1.26 (0.58 to 2.72)	0.560
As a GP, I have been involved in handling the patient' anxiety and psychological concerns	124	0.44 (0.21 to 0.92)	0.029	0.64 (0.31 to 1.35)	0.245
As a GP, I found the information from the department to help me manage the physical issues	116	0.88 (0.41 to 1.88)	0.739	1.55 (0.70 to 3.47)	0.283
As a GP, how satisfied are you, with the distribution of tasks and roles regarding initiatives practice could initiate concerning the patient trajectory?	117	3.03 (1.35 to 6.82)	0.007	4.14 (1.62 to 10.56)	0.003
As a GP, how satisfied are you, with the distribution of tasks and roles of who should take care of side-effects and late complications?	121	1.34 (0.57 to 3.20)	0.504	1.49 (0.60 to 3.70)	0.389

^a^OR >1 favour the intervention, meaning the GP being more involved, information being more helpful, or the GP being more satisfied with suggestion and clarification of responsibility. OR = odds ratio.

Two items showed insignificantly increased OR: *‘As a GP, I have been involved in the treatment of comorbidity* *‘* and *‘As a GP, how satisfied are you, with the distribution of tasks and roles of who should take care of side-effects and late complications?* *‘*; while insignificantly decreased OR was observed for *‘As a GP, I found the information from the department helpful in managing the physical issues* *‘* (Table 3).

## Discussion

### Summary

The evaluation of the PSP randomised controlled trial showed that involving the GP in one consultation with the cancer patient and oncologist could significantly enhance the OR for the GPs being satisfied with the distribution of tasks and roles between sectors, and feeling well informed regarding how to support the cancer patient. This new way of shared care was compared with usual care. These results also suggested less involvement of GPs in handling the patients' anxiety and psychological problems. Recruitment and response rates from the GPs were challenging, with a lower than expected total response rate of 44%, which should be considered when interpreting the results.

The result suggesting a mechanism of decreased involvement in anxiety and psychological issues by the GP did not support the authors' hypothesis. The decrease could either be an artefact, or perhaps patients seeing both doctors communicating gave them more peace of mind, thereby reducing the anxiety and leading to less involvement from the GP.

### Strengths and limitations

The current study has several limitations to be considered. Sample bias due to a challenging recruitment process of patients, and subsequently their GPs, lead to the low overall response rate. Even though GPs are often challenging to recruit for research studies,^27–29^ the response rate in this study was considered lower than expected.^30^ These numbers, and the fact that GPs with a greater interest in cancer patients may be more likely to respond, might give room for selection bias. Due to the COVID-19 pandemic, the authors were not able to send the survey to the last ten GPs and response rate could have potentially been 5% higher.

This study did not use a validated questionnaire. No relevant questionnaire existed, and a rigorous validation process was not prioritised in the development phase of the trial. However, the definition of themes and items were based on the literature.^15,31–33^ Formulations and face validity was tested by feedback from a user panel of GPs and oncologists during piloting.^22^ Moreover, the authors minimised the risk of overestimating the effect of the study by reducing the number of items for between-group analysis.

The study might be diminished in power. The sample size calculations did not refer to the secondary outcomes reported in this study. Furthermore, the numerous technical problems resulted in cancellation or alternative ways of tripartite communication in 32% of the consultations planned.

Finally, it is unknown whether any spill-over effects may have improved care for the patients in the control group, leading to a smaller impact of the intervention. To minimise the spill-over risk, the authors did not inform GPs about their patients being involved in the study if enrolled in the control group before they received the questionnaire. Although their patients may have been told about the target of the study, GPs were in some way blind. However, the blinding of patients and GPs in the intervention group was impossible. Only nine of 105 GPs had >1 patient in the study, and eight had patients in both groups. Therefore, it is believed that the risk of spill-over was minimal.

The study had the advantage of being randomised. The study included patients with various cancer types, different prognosis, health problems, and needs of supportive care. However, lung and gastrointestinal cancer were in the majority. The patient heterogeneity of the study mimics the patient population in the department as well as general practice in the Region,^34^ which is a strength for generalisability and future implementation.

Finally, including intention-to-treat analysis reflects on everyday clinical practice and enhances external validity.^35,36^ The results of the intention-to-treat and per-protocol analysis showed similar results, and the authors wonder if the introduction of the concept of cooperation between sectors and the overall goal of involving the GP, independent of the completion of the video consultation, could be a substantial component.

### Comparison with existing literature

The high OR for direct contact indicates that the GPs recall the video consultations and found it useful, as indicated in the process evaluation of the trial.^26^ During process evaluations, it was shown that the consultations succeeded in being patient-centred, could be managed within daily clinical routines, and improved the coordination of care as perceived by patients, oncologists, and GPs.^24,26^ In line with a review about shared care models, stating that involving GPs and improving communication between sectors can benefit cancer care, this study indicates enhanced GPs involvement and, furthermore, that the GPs were satisfied with the distribution of tasks.^10^

A Cochrane review on shared care models across sectors suggests that shared care may have the potential to provide long-term benefit for patients with depression and increase effectiveness of the organisation of shared care if introduced at an early stage in the disease process using information technology.^11^ Moreover, the review states that shared care model should be develop for other conditions. The PSP used video consultation at an early stage in the trajectory. Introducing sharing and the use of information technology is believed to be an essential mechanism for the study effect on GPs involvement.

Improving communication between primary and secondary care can lead to an increase in GP involvement.^37^ Higher GP involvement calls for easy access to sufficient knowledge.^38^ A shift from one-way communication in summary letters to real-time dialogue may be important for improving communication and involvement. This study has shown that arranging direct contact could increase the GP’s perception of involvement in the trajectory and lead to a more precise distribution of tasks and roles.

### Implications for research and practice

Despite compromised participation rates and technical challenges, this study showed that communication by bringing health professionals and patients together by video was perceived as useful, and resulted in GPs feeling better informed about their role. These results are believed to have a positive impact on the recruitment of patients and health professionals for the next generation of PSP.

The partnership intervention makes a difference at the physician level and supports the theories of the mechanisms of this shared care programme.^22^ Assuming reliable IT solutions were available, the authors concluded that with relatively limited effort, the sharing of 15 minutes through video across sectors as part of a routine communication can help GPs in providing better care for cancer patients.^26^ The COVID-19 pandemic may have increased the willingness to participate in video consultations dramatically.

Future research should focus on the need for a validated questionnaire to measure GPs' experiences with tripartite video consultations. The questionnaire in this study could be used as a template. Moreover, investigations on the patient–physician relationship, when GPs declined to participate in a study like this, are welcomed.

Finally, the evaluation of patient outcomes is crucial before implementation into routine care. Patient evaluation of coordination of care and health-related quality of life are among planned outcomes.
